# Correction: *What, how, when and who of trial results summaries for trial participants: stakeholder-informed guidance from the RECAP project*

**DOI:** 10.1136/bmjopen-2021-057019corr1

**Published:** 2023-12-13

Bruhn H, Campbell M, Entwistle V, et al. What, how, when and who of trial results summaries for trial participants: stakeholder-informed guidance from the RECAP project *BMJ Open* 2022;12:e057019. doi: 10.1136/bmjopen-2021-0570198687

This article was previously published with an error.

The same image was used for figures 1 and 2. Figure 1 has now been corrected.

**Figure 1:**
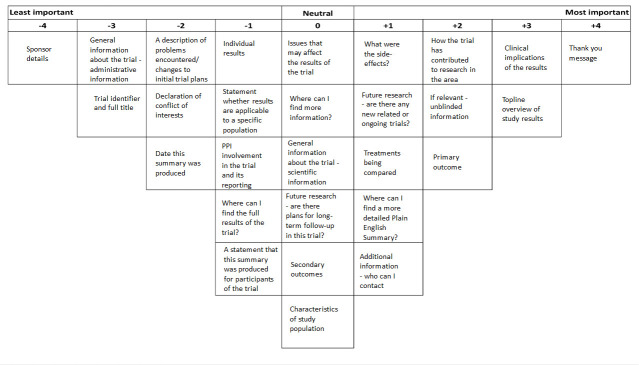
Viewpoint 1 Q-sort: ‘population view’. PPI, patient and public involvement.

