# Supplementary material for: What, how, when and who of trial results summaries for trial participants: stakeholder-informed guidance from the RECAP project
Source: BMJ Open. 2022 Mar 24;12(3):e057019. doi: 10.1136/bmjopen-2021-057019 (PMC8961145; doi:10.1136/bmjopen-2021-057019)
Supplement: Supplementary data [file bmjopen-2021-057019supp001.pdf]

Supplementary Table 1. Stakeholder groups and approaches to recruitment.

Stakeholder group	Recruited through
Ethics committee members from NHS research ethics committees	-Health Research Authority (HRA), UK
Trialists working in Clinical Trial Units (including Trial Managers)	-UK Clinical Research Collaboration Clinical Trials Units<br>-study team's personal contacts
Sponsors (e.g. NHS, Academia, industry)	-NHS Research & Development Forum<br>-Association of Research Managers and Administrators (ARMA)
Research Funders	-ARMA<br>-Direct contact to funders of Phase III pragmatic trials in the UK
Participants from ongoing and recently completed trials	-Eligible ongoing UK wide trials in The Centre for Healthcare Randomised Trials (CHaRT), University of Aberdeen
Members of the public	-SHARE
Regulators	-HRA<br>-National Institute for Health and Care Excellence<br>- Medicines and Healthcare products Regulatory Agency

Supplementary Table 2. Q-methodology Concourse

Resource	Link
Statements from individuals on Health Talk talking about provision of trial results.	https://www.healthtalk.org/clinical-trials/feedback-of-trial-results
Center for Information and Study on Clinical Research Participation (CISCRP)	https://www.ciscrp.org/services/health-communication-services/trial-result-summaries/
Summaries of Clinical Trial Results for Laypersons Recommendations, version 2, 5 February 2018<br><br>Recommendations of the expert group on clinical trials for the implementation of Regulation (EU) No 536/2014 on clinical trials on medicinal products for human use<br>-Annex 1 – Templates with example wording	https://ec.europa.eu/health/sites/default/files/files/eudralex/vol-10/2017_01_26_summaries_of_ct_results_for_laypersons.pdf
EPF position: Clinical trial results – communication of the lay summary, 02/03/2015	https://www.eu-patient.eu/globalassets/policy/clinicaltrials/epf-lay-summary-position-final_external.pdf
EPF's response to the European Commission's public consultation on the "Summary of Clinical Trial Results for Laypersons", August 2016	https://www.eu-patient.eu/globalassets/policy/clinicaltrials/epf-response-to-the-lay-summary-public-consultation--august-2016.pdf
Summary of clinical trial results for laypersons – August 2016<br>Joint response from Cancer Research UK and the British Heart Foundation	https://ec.europa.eu/health/sites/default/files/files/clinicaltrials/2016_06_pc_guidelines/gl_3_resp_cancer_res_uk_british_heart_found.pdf
MRCT Center Return of Aggregate Results to Participants Guidance Document, version 3.1, 22 November 2017<br>-4.3 Essential Sections for Plain LanguageSummaries(PLS)	https://mrctcenter.org/wp-content/uploads/2017/12/2017-12-07-MRCT-Return-of-Aggregate-Results-Guidance-Document-3.1.pdf
MRCT CenterReturn of Individual Results to Participants Recommendations Document, version 1.2, 22 November 2017<br>-6.1WhatShould be Returned?	https://mrctcenter.org/wp-content/uploads/2017/12/2017-12-07-Return-of-Individual-Resullts-Recommendations-Document-V-1.2.pdf
Draft FDA Guidance on Provisio nof Plain Language Summaries, 13/06/2017<br>Issued by: Center for Drug Evaluation and Research, Center for Biologics Evaluation and Research, and Center for Devices and Radiological Health	https://mrctcenter.org/wp-content/uploads/2017/06/2017-06-13-MRCT-Draft-FDA-Guidance-Return-of-Aggregate-Results.pdf
EFA response to the recommendations of the expert group on clinical trials on the summary of results for laypersons: the perspective of patients with allergy, asthma, and chronic obstructive pulmonary disease, June 2016	https://ec.europa.eu/health/sites/default/files/files/clinicaltrials/2016_06_pc_guidelines/gl_3_resp_efa.pdf
Summary of the responses to the public consultation on "summaries of clinical trial results for laypersons" recommendations of the expert group on clinical trials for the implementation of regulation (eu) no 536/2014 on clinical trials on medicinal products for human use	https://ec.europa.eu/health/sites/default/files/files/clinicaltrials/2016_06_pc_guidelines/gl_3_summary.pdf
REFLECTION PAPER --- EFPIA Guiding Principles on Layperson Summary EU Clinical Trials Regulation 536/2014 – Annex V	https://www.efpia.eu/media/25661/reflection-paper-efpia-guiding-principles-on-layperson-summary.pdf

Supplementary Table 3. Q-sort vignettes

Vignettes for members of the public with and without trial experience

Imagine you have participated in a clinical trial. The trial was run in the real-life setting of the NHS (this is also called a pragmatic effectiveness trial) and aimed to establish what would work under 'normal' conditions. In the trial two treatments were compared. One group received the new treatment and another group received the treatment that the new treatment was being compared to. You and the other participants were assigned to a group by chance and did not know which group you were in.

1) The trial is now finished and the findings show that the new treatment was more effective than the treatment it was being compared to.

What information would be important to you to include in the results provided to trial participants such as yourself?

Imagine you have participated in a clinical trial. The trial was run in the real-life setting of the NHS (this is also called a pragmatic effectiveness trial) and aimed to establish what would work under 'normal' conditions. In the trial two treatments were compared. One group received the new treatment and another group received the treatment that the new treatment was being compared to. You and the other participants were assigned to a group by chance and did not know which group you were in.

2) The trial is now finished and the findings show that the new treatment was as effective as the treatment it was being compared to.

Imagine you have participated in a clinical trial. The trial was run in the real-life setting of the NHS (this is also called a pragmatic effectiveness trial) and aimed to establish what would work under 'normal' conditions. In the trial two treatments were compared. One group received the new treatment and another group received the treatment that the new treatment was being compared to. You and the other participants were assigned to a group by chance and did not know which group you were in.

3) The trial is now finished and the findings show that the new treatment was not as effective as the treatment it was being compared to.

Vignettes for professionals (REC members, funders, sponsors, trialists etc.)

A clinical trial has been conducted. The trial was run in the real-life setting of the NHS (this is also called a pragmatic effectiveness trial) and aimed to establish what would work under 'normal' conditions. In the trial two treatments were compared. One group received the new treatment and another group received the treatment that the new treatment was being compared to. Trial participants were assigned to a group by chance and did not know which group they were in.

1) The trial is now finished and the findings show that the new treatment was more effective than the treatment it was being compared to.

A clinical trial has been conducted. The trial was run in the real-life setting of the NHS (this is also called a pragmatic effectiveness trial) and aimed to establish what would work under 'normal' conditions. In the trial two treatments were compared. One group received the new treatment and another group received the treatment that the new treatment was being compared to. Trial participants were assigned to a group by chance and did not know which group they were in.

2) The trial is now finished and the findings show that the new treatment was as effective as the treatment it was being compared to.

A clinical trial has been conducted. The trial was run in the real-life setting of the NHS (this is also called a pragmatic effectiveness trial) and aimed to establish what would work under 'normal' conditions. In the trial two treatments were compared. One group received the new treatment and another group received the treatment that the new treatment was being compared to. Trial participants were assigned to a group by chance and did not know which group they were in.

3) The trial is now finished and the findings show that the new treatment was not as effective as the treatment it was being compared to.

Supplementary Table 4. Per protocol stakeholder groups loading onto each factor.

Per protocol stakeholder group	Factor 1 Population n = 19 (60%)	Factor 2 Individual n = 13 (40%)
PPI members	1[†]	4[†]
Members of the public with clinical trial experience	0	5
REC members	6	1
Clinical trial funding bodies representatives	4	0
Sponsor representatives	2	2
Regulatory representatives	1	1
CTU staff/trialists	5	0

[†] Participants may have been identified through funders or regulators but had role of PPI Partner within that organisation.

Supplementary Table 5. Content analysis of result summaries

#	Q-set item	Present (n/%)
1	Trial identifier and full title	12 (40%)
2	Topline overview of study results	8 (27%)
3	Sponsor details	8 (27%)
4	Declaration of conflict of interests	0 (0%)
5	General information about the trial - administrative information	17 (57%)
6	General information about the trial - scientific information	19 (63%)
7	Characteristics of study population	13 (43%)
8	Treatments being compared	29 (97%)
9	What were the side-effects?	14 (47%)
10	Primary outcome	30 (100%)
11	Secondary outcomes	25 (83%)
12	Statement whether results are applicable to a specific population	13 (43%)
13	Issues that may affect the results of the trial	0 (0%)
14	How the trial has contributed to research in the area	17 (57%)
15	Future research - are there plans for long-term follow-up in this trial?	5 (17%)
16	Future research - are there any new related or ongoing trials?	9 (30%)
17	Additional information - who can I contact	19 (63%)

18	Where can I find more information?	17 (57%)
19	Where can I find the full results of the trial?	15 (0%)
20	Individual results	0 (0%)
21	If relevant - unblinded information	1 (3%)
22	Thank you message	26 (87%)
23	PPI involvement in the trial and its reporting	3 (10%)
24	Date this summary was produced	15 (50%)
25	A description of problems encountered/changes to initial trial plans	1 (3%)
26	A statement that this summary was produced for participants of the trial	11 (37%)
27	Clinical implications of the results	13 (43%)
28	Where can I find a more detailed Plain English Summary?	1 (3%)
**Other items**		
i	Funder	11 (37%)
ii	Cost	9 (30%)
iii	Content – questionnaire data	5 (17%)
iv	What do these results mean for you	4 (13%)

Supplementary Table 6. Focus group exemplar quotes to support themes

Theme	Exemplar quotes
Experiences of current practice when sharing trial results summaries with participants	*'….informing participants of results needs to be part of the plan early on doesn't it because you know, if we can think about what journal we want to put it in and what conference we want to go to then we can think about how we're going to actually engage with the people that have made the study possible.'*<br><br>*'I've worked in research for years in different diseases and we've never, ever been upfront about… I don't think there's any particular collusion, I just don't think it's occurred to us to make you know, those robust processes or what happens when the trial is finished and reported as we do when we're consenting and treating.'*<br><br>*'….a bit we talked about is that contracts end, trials end, the money ends, the person leaves and nobody's there to do that and it just doesn't get handed over, whereas industry have got the luxury of probably a designated person that does'*<br><br>*'but when you look at a lot of studies, not the commercial studies, when looked at the other studies, the academic studies, the ones which are run by universities where the need is from universities, his or her employment is based on how many grants they get in and how many journal articles they put out and how many journal articles are in high impact journals. They don't get any points at all for the number of, and forgive me if I'm speaking out of turn because I'm NHS…*<br>*R2 - No, it's true.*<br>*FP - … but they don't get any additional points for having made sure all the registrations up to date, having made sure they've published the protocol paper, having made sure every single participant has been engaged in receiving results. '*<br><br>*'I mean I'm thinking with my funder hat on now, actually we give money for you know, people ask us for money and we give them the money and then once that relationship's stopped we stop caring because we have no contract, and we stop giving the money so they won't do the trial. Also another bit whereas if it was in from the beginning and actually maybe you don't give a final payment*

	until we're really confident that you've done that piece of engagement then and I hate it, I hate to talk about leverage, but actually sometimes you just have to…'<br>*yes, as a drug study, there's a legal imperative. For non-drug studies though, for device studies and other intervention studies, there isn't a legal imperative, although the HRA are bringing in some fairly strict guidance and the MIHR are now making it a requirement that results are uploaded to open access registries within 24 months.'*<br><br>*'It's the thing that the university clinician with you in the study is being scored on. It's not, how do you make sure every single one of your patients has been engaged? How do you ensure every single one of your patients has been thanked? Have you made sure that they've all received it? It's, have you made sure your journal is in the New England Journal of Medicine? So there is something about that cultural bit that we desperately need to address. Not in… as a nurse by background, I completely agree with the fact that we do pander to… that side is frustrating and at the moment, unnecessarily '*<br><br>*'I mean I've done four trials okay, admittedly two of them are still ongoing, but on the other two I know they're finished and I've had no feedback whatsoever as to what's happened.'*<br>*'but it's not necessarily going to help you as a patient but you will be helping the future generations. Well if we're going to sign up for this and help future generations, why are we forgotten about?'*<br><br>*'So unless you're actually building your comms into the design of your study, then it's not going to help because you're not going to employ the hours to allow somebody within the research team to do it as a valid part of the obligation to do research.'*
What methods should trial teams use to share results with trial participants?	*'…..a decent comms plan should be costed into a study so the funders have an obligation to fund that aspect, and it should be properly planned and again appropriate to the group.'*

	'The word that keeps on popping up throughout that I've underlined is "personalised" and "personal". Also, asking people what they want to be told and how they want to be told it, you know, actually one size does not and will not ever fit all. This mixed messages, sort of layered approach seems to be a repeated theme throughout what we've been discussing.' <br>*'It's going to depend on what the research is. If the research is for multi-centre trials, for dangerous drugs or fatal conditions, it's terribly different to people whether a behaviour therapy is going to help depression. And if you're talking about quite specific physical things like, "Do drugs work?" it's very different to a questionnaire surveys about people's attitudes. And I think that the different modes that you put up there would be appropriate to different methods of research.'* <br>*'A lot of people are not computer literate, especially the older generation. I mean I could take you down to Cornwall and the amount of people who say, "Well I haven't got a computer".'* <br>*'we've certainly had that at our trust when we do the Parkinson's study and the patients had to invest very, very heavily in this study because they had to have surgery and then the drug didn't work and how… breaking that news to people, some of whom genuinely felt they had benefited. So there was this disparity between how individuals find the trial results, that was a really difficult conversation for the chief investigator to have. '* <br>*'I know we're talking ideal world, but I have spoken to people in clinical trials and what I – this is very controversial, but what I really want to do is be able to talk to other people who've been in the same position. What they wanted was a support group.* *So like a forum?* *A peer support group. Yes. I know that comes up with sorts of problems with confidentiality and god knows what else, but people are really keen on… I never thought about it, but I thought, yes, it might answer some of the questions and also make us all feel part of it…'*

When should results summaries be shared with trial participants?	*'They should have the opportunity to have the information, and if they don't want it at that moment, they should be told that they can ask for it when they're ready'*<br><br>*'But also, just looking at the second part of when you ask people what it is they want to find out about, you've got to consider that not everybody manages to complete a trial. That might not be because of an adverse effect, that might be because something else happens and they have to withdraw. They could be really disappointed that they've had to withdraw, but they would still be really interested in knowing when it's complete, when the analysis is being done, when the findings available. So, from the point of view of asking them, you've got to ask that at the beginning because you might not be happy….I would go further, I say you should ask at the beginning and then you should also ask when they've finished their research visits. Whether that's because they want to come off whatever, or because they've finished or whatever. I think the last conversation you should have should be, "So at the beginning you wanted to know X, Y, Z, is that still the case?"'*<br><br>*'we kind of came up with the expectations and initial consent to receiving information right at the start of the study, but then also repeating at the end of your involvement, whether that's imminently because the results are about to come out or actually… my involvement in the study is finished now but we actually know it's going to go on for another five years. So, that last contact…'*<br><br>*'All that's got to become more visible, so why wouldn't we actually put a kind of data sharing focus on studies right from the beginning, it's my data, this study has been successful because you used information about me and I gave you my permission to use it, and now that you've used it, you've got your answer, I'm the first person I should know.'*<br><br>*'We try to ensure that the information for the participants is ready to be released at the same time, and yes, we've not been able to do that by hand. And I don't know many journals that will let you change that. It's also some people get questioned from, sorry, particularly if it's a results that the whole institution thinks is important, you get more pressure from relations and marketing to actually have a wider audience which you don't need to send(?) off(?) control, in a way. You can give the information that you think*

is appropriate, but how, say newspapers, and other media outlets, beyond that, it's completely beyond your dynamics(?) in a way that it's not helpful. I don't know how to word it.
F1 – And the embargo is usually placed by the journal, but do you think as well that maybe we need to be talking to funders about…and journals? '

'I think the researchers want it out as soon as they can as well. I guess the question is really, whether… if you're unlucky enough to go through nine journals or what-have-you, then when should you tell patients? Should you tell patients when you hit the registry deadline or should patients hear about it before it goes public? '

'Let's say it goes to peer review, you've submitted your manuscript, at that point you then work with your patient group to think about how can we translate this into a meaningful message to those who participated. And then we try to line it up so that, when we know that, you know, on the 12th August that the paper is going to be published, that on the 12th August, and if we can, we've let people know, on the 12th August, we're going to let you know what the results are, and we can send them a link to the journal as well, but that however we decide to send it.'

'FP – But practically, I don't think you can ever get… you can't give a patient a result as soon as they finish, and that should be fully transparent at the beginning that you're going to get the results when everyone has finished. Whether that's five years, ten years, whatever it is.
FP – Yeah, you should have that understanding, before this timeline. But yeah, you should acknowledge your participation and say, "We anticipate the final patient completing in 20 months' time," and then you should write to them again and say, "Everyone has finished, we're now back towards analysis," then say when the analysis is complete, so you have constant information'.

'"transparent", "personalised", "considered", you know, all those sort of things. It kind of comes back to that really, doesn't it? So you might say, "Results are ready, this is a thank-you letter to everybody to say the results are ready," but the personalised bit might have to come… "We might send you something more personalised because we only have this timeframe…"
FP – So we'll actually sometimes do it the other way around. We generally might have unblinded the trial sooner because the analysis is being formed, so we'll know who was on A or B or placebo, and we can tell the patients that and we can say, "This trial is being analysed, the results will be here on this day," or whatever. Then we're doing the personal bit as soon as we can in terms of telling people.'

'FP – So, personalised, meaning individual results? So results just for the person?
MP – Yes, some personal indicator. I can see an argument where you clinically are a clinician, it might be more personal but it's after the global bit. You hear them say, "This drug can do X, Y and Z," and then you say, "This is what it did to you.
FP - We informed them by asking their… because it was easier to manage. And usually they had a research nurse, and because it was a specialist oncology centre, they were specialists and there were other support people there that would be able to talk to them. So for the ones that were on the drug that wasn't quite as effective, they were told personally before the press release came out.'

'When you sign an information sheet, you often say, "I won't go blabbing about this trial to the world," so why can't you just say, "I won't reveal…" and again, if it was your individual results, it's not going to affect the publication or results if you tell the individual. As I say, we often tell people if they were on A or B earlier, then we tell them the trial results.'

'Once you know that you absolutely know what you think you know! You don't want to tell people the wrong thing because that's not entirely helpful. '

	'so if you're actually able to reassure us at the beginning of a study that we're going to collect your personal health information, we're going to use it for this particular reason, and at the earliest opportunity we will tell you what we've found out, that must be a good principle, you know? The earliest opportunity would be around, is it safe to give the patient community this information now?'
What else should trial teams consider when sharing results	*'think what's really interesting is there seems to be two places where the buck is stopping: the local level and then the sponsor level. So every single research trial has to have a sponsor, they are the people, the chief investigators who take the responsibility for the entire trial. Although they may not know the participants as individuals, the responsibility still falls there. What we're talking about here is a lot of this is all about your local level, so should there be another level, another back-up level, which is beyond what you get at your personal site, there should be something that the sponsor site is providing that supports you through this. Is there something that maybe isn't quite as personalised or someone you could go and talk to that should be provided by… they'll always be there because they're writing up the results, they're not just going to disappear, whereas nurses, of course, as you said, funding… they move on, the structures change. Is there something we could identify, like a golden rule telephone number, or something like that?'*
How do we know we have done it well?	*'Would you take part in research more because of the information you received? I think that's the crux of it, we're trying to create a community that is a research community and this is the end of it.. '*
